# Relationships of nuclear, architectural and International Federation of Gynecology and Obstetrics grading systems in endometrial cancer

**DOI:** 10.4274/jtgga.2017.0004

**Published:** 2018-03-01

**Authors:** Tayfun Toptaş, Elif Peştereli, Selen Bozkurt, Gülgün Erdoğan, Tayup Şimşek

**Affiliations:** 1 Clinic of Gynecologic Oncology, University of Health Sciences, Antalya Training and Research Hospital, Antalya, Turkey; 2 Department of Gynecopathology, Akdeniz University School of Medicine, Antalya, Turkey; 3 Department of Biostatistics and Medical Informatics, Akdeniz University School of Medicine, Antalya, Turkey; 4 Department of Gynecologic Oncology, Akdeniz University School of Medicine, Antalya, Turkey

**Keywords:** Endometrial cancer, grade, lymph node involvement

## Abstract

**Objective:**

To examine correlations among nuclear, architectural, and International Federation of Gynecology and Obstetrics (FIGO) grading systems, and their relationships with lymph node (LN) involvement in endometrioid endometrial cancer.

**Material and Methods:**

Histopathology slides of 135 consecutive patients were reviewed with respect to tumor grade and LN metastasis. Notable nuclear atypia was defined as grade 3 nuclei. FIGO grade was established by raising the architectural grade (AG) by one grade when the tumor was composed of cells with nuclear grade (NG) 3. Correlations between the grading systems were analyzed using Spearman’s rank correlation coefficients, and relationships of grading systems with LN involvement were assessed using logistic regression analysis.

**Results:**

Correlation analysis revealed a significant and strongly positive relationship between FIGO and architectural grading systems (r=0.885, p=0.001); however, correlations of nuclear grading with the architectural (r=0.535, p=0.165) and FIGO grading systems (r=0.589, p=0.082) were moderate and statistically non-significant. Twenty-five (18.5%) patients had LN metastasis. LN involvement rates differed significantly between tumors with AG 1 and those with AG 2, and tumors with FIGO grade 1 and those with FIGO grade 2. In contrast, although the difference in LN involvement rates failed to reach statistical significance between tumors with NG 1 and those with NG 2, it was significant between NG 2 and NG 3 (p=0.042). Although all three grading systems were associated with LN involvement in univariate analyses, an independent relationship could not be established after adjustment for other confounders in multivariate analysis.

**Conclusion:**

Nuclear grading is significantly correlated with neither architectural nor FIGO grading systems. The differences in LN involvement rates in the nuclear grading system reach significance only in the setting of tumor cells with NG 3; however, none of the grading systems was an independent predictor of LN involvement.

## Introduction

Endometrioid-type endometrial cancer (EC) is graded histologically according to the criteria set forth by the International Federation of Gynecology and Obstetrics (FIGO) (1). This grading system consists of a combination of two different grading systems, architectural grading and nuclear grading. In the FIGO grading system, features for architectural grading have been adopted from well-defined criteria of the Gynecologic Oncology Group (GOG) pathology committee (2). FIGO stated that in tumors with notable nuclear atypia that is inappropriate for the architectural grade (AG), the final grade should be established by raising the AG by one grade (3). However, FIGO did not define any criteria to determine “notable nuclear atypia”, which led to confusion both for pathologists and physicians.

Lymph node (LN) involvement is one of the main prognostic factors for patients with EC. The five-year overall survival rate exceeds 80% in patients with negative LNs, but in cases of LN metastasis, it decreases to approximately 50% (3). Several primary tumor characteristics have been demonstrated to be related with the risk of LN metastasis, of which tumor grade is one of the most consistently reported. 

In the present study, by using strict diagnostic criteria, we aimed to examine correlations among the nuclear, architectural, and FIGO grading systems, and their relationships with LN involvement in endometrioid-type EC.

## Material and Methods

The clinicopathologic records of patients with EC, who underwent total hysterectomy and systematic pelvic lymphadenectomy with or without paraaortic LN dissection at a single institution between January 2010 and January 2015, were reviewed retrospectively. Patients with non-endometrioid histotype, primary synchronous malignancy, no residual disease in the hysterectomy specimen, or who had not undergone LN dissection were excluded.

As a routine strategy at our institution, all patients with newly diagnosed EC were offered treatment with total hysterectomy with systematic pelvic lymphadenectomy if they were medically operable and did not desire fertility preservation. Paraaortic LN dissection was added to pelvic lymphadenectomy in the presence of at least one of the following risk factors: a) non-endometrioid histotype, b) FIGO grade 2 or 3 endometrioid carcinoma, c) deep (≥50%) myometrial invasion on frozen-section examination.

The study was performed in accordance with the ethical standards described in an appropriate version of the 1975 Declaration of Helsinki, as revised in 2013. Written informed consent was not required for this type of retrospective study. Ethical approval was obtained from the institutional local ethics committee.

All available histopathology slides were reviewed in each case by two sub-specialized gynecologic pathologists with respect to primary tumor characteristics including histotype, AG, nuclear grade (NG), FIGO grade, and LN involvement. 

Architectural grading was performed using the criteria of the GOG pathology committee (2): AG 1, tumors with well-preserved glandular morphology in which solid nests of neoplastic cells comprise ≤5% of the lesion; AG 2, tumors in which the solid areas comprise 5 to 50% of the lesion; and AG 3, tumors in which >50% of the lesion is arranged in solid sheets of neoplastic cells. AG was based upon assessment of glandular and solid areas, excluding areas of squamous differentiation.

Nuclear grading was performed using the criteria defined by Zaino et al. (4): NG 1, uniform round-to-oval nuclei, with even distribution of chromatin and inconspicuous nucleoli; NG 2, irregular oval nuclei, with chromatin clumping and moderate size nucleoli; and NG 3, large, pleomorphic nuclei, with coarse chromatin, and large irregular nucleoli. NG of a tumor was assigned based on the features displayed by the majority of tumor cells.

**Figure 1:**
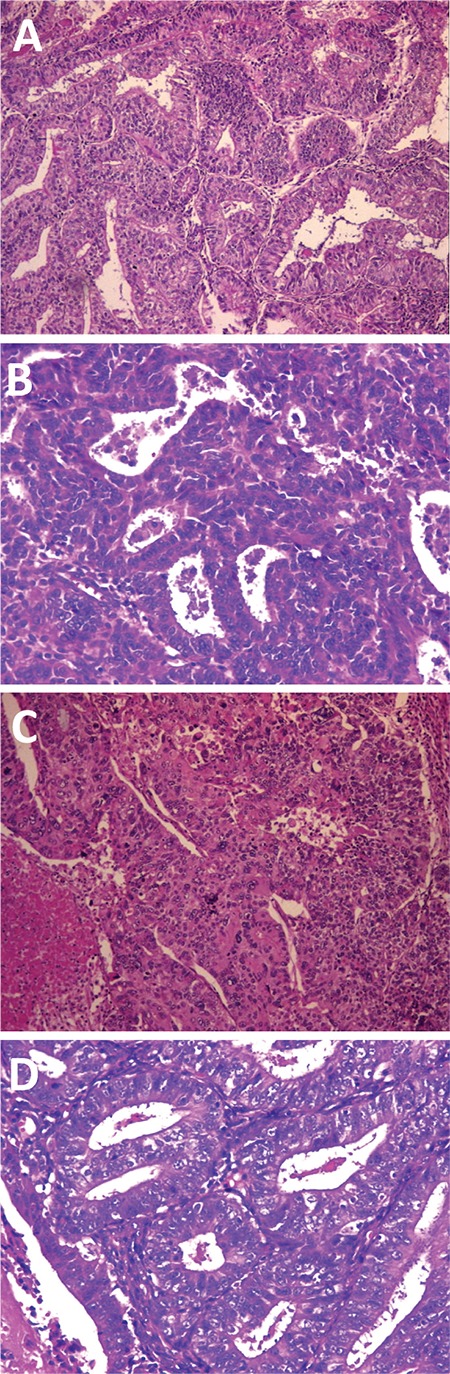
Microscopic views of samples from each grading systems; a) FIGO grade 1: composed of AG 1 and NG 1 (Hematoxylin & Eosin, x100), b) FIGO grade 2: composed of AG 2 and NG 2 (Hematoxylin & Eosin, x200), c) FIGO grade 3: composed of AG 3 and NG 3 (Hematoxylin & Eosin, x100), d) FIGO grade 2: consisting of cells with “notable nuclear atypia (NG 3)” inappropriate for the architectural grade (AG 1) (Hematoxylin & Eosin, x200) NG: Nuclear grade; AG: Architectural grade; FIGO: International Federation of Gynecology and Obstetrics

In the present study, the “notable nuclear atypia” was defined as NG 3, and the FIGO grade was established by raising the AG by one grade when the tumor was composed of cells with NG 3. Figure 1 shows microscopy views of the samples from each grading system.

### Statistical analysis

Statistical analyses were performed using IBM SPSS Statistics 20 (SPSS/IBM, Chicago, IL, USA) software. Correlations between the grading systems were analyzed using Spearman’s rank correlation coefficient. The relationships of primary tumor characteristics with LN involvement were assessed using logistic regression analysis. Variables with a p value <0.05 in univariate analysis were included into multivariate analysis. The effects of variables on LN involvement were reported as adjusted odds ratios and 95% confidential intervals. 

## Results

A total of 135 patients were enrolled in the analysis. The majority of patients had AG 1 (56.3%), NG 2 (45.9%), and FIGO grade 1 (54.1%) tumors. Eighty (59.3%) patients had pelvic lymphadenectomy alone, and 55 (40.7%) had combined pelvic and paraaortic lymphadenectomy. LN involvement was identified in 25 (18.5%) patients (Table 1).

Correlation analysis revealed that there was a significant and very strongly positive relationship between the FIGO and architectural grading systems (r=0.885, p=0.001); however, correlations of nuclear grading with the architectural (r=0.535, p=0.165) and FIGO grading systems (r=0.589, p=0.082) were moderate and statistically non-significant (Table 2).

The rates of LN involvement according to each grading system are summarized in Table 3. LN involvement was detected in 7.9% of tumors with AG 1, 25.0% of tumors with AG 2, and 47.3% of tumors with AG 3. LN involvement rates according to FIGO grades were as follows: 5.4% for grade 1, 31.6% for grade 2, and 37.5% for grade 3. LN involvement rates differed significantly between tumors with AG 1 and AG 2 (p=0.045), and between tumors with FIGO grades 1 and 2 (p=0.031), whereas there were no significant differences between AG 2 and AG 3 (p=0.069), and between FIGO grades 2 and 3 (p=0.327).

The rates of LN involvement based on nuclear grading system were as follows: 6.2% for NG 1, 20.9% for NG 2, and 36.0% for NG 3. In contrast to architectural and FIGO grading systems, the difference in LN involvement rates failed to reach statistical significance between tumors with NG 1 and those with NG 2 (p=0.115), but it was significant between NG 2 and NG 3 (p=0.042) (Table 3).

In order to assess independent relationships between grading systems and LN metastasis, two different logistic regression models were developed because a strong correlation between FIGO grade and AG would confound the possible associations (Table 4). NG and AG were assigned to the first model, and the FIGO grade was separately evaluated in the second model. Both models also included deep myometrial invasion and lymphovascular space involvement (LVSI) as potential covariates. Although all three grading systems were associated with LN involvement in univariate analyses, an independent relationship could not be established after adjustment for other confounders in multivariate analyses. LVSI was consistently the sole independent predictor of LN metastasis in multivariate analyses (p=0.001).

## Discussion

After FIGO’s equivocal statement regarding nuclear atypia, some researchers attempted to develop more objective definitions in nuclear as well as final grading of EC. First, Zaino et al. (4) reported that if the “notable nuclear atypia“ was defined as grade 3 nuclei, and the final FIGO grade was established by raising the AG by one grade only when the majority of the neoplasm was composed of cells with NG 3, the FIGO grading system showed prognostic utility. Following the analysis of the clinicopathologic data obtained from 715 patients with endometrioid EC, the authors found that tumors upgraded using this criterion had a relative risk of progression 1.9 times higher than that of the group from which they were moved. In contrast, if the notable atypia was considered as both NG 2 and NG 3, the relative risk was almost identical to that of the group from which they were moved. Later, in a study of 476 patients with endometrioid EC, Takeshima et al. (5) suggested that upgrading of AG should be performed when more than 25% of the neoplastic cells showed grade 3 nuclei. The authors reported that tumors that had 26% to 50% of neoplastic cells with grade 3 nuclei showed a similar risk of recurrence as did tumors that had more than 50%. In a large single institutional analysis that investigated a convenient method for the modification of AG by nuclear features, Ayhan et al. (6) reported that in determining the FIGO grade, upgrading of AG 1 or AG 2 tumors by grade 3 nuclei was the most reliable method. The authors also noted that all three grading systems significantly predicted poor disease outcome, but only the FIGO grade, stage, and cervical involvement remained independent predictors of survival in multivariate analysis.

Our data indicated that tumors with grade 3 nuclei significantly differed from tumors with NG 1 and from NG 2 in terms of LN involvement. On the contrary, such a significant difference was not evident between tumors with NG 1 and those with NG 2. These findings support previous studies (4,5,6) that suggested that “notable nuclear atypia” should be defined as NG 3.

The lack of an objective definition for “notable nuclear atypia” and the moderate inter-observer agreement in distinction of squamous from non-squamous solid growth in the FIGO grading system led to the proposal of alternative binary grading systems by some researchers over the past two decades (7,8,9). Lax et al. (7) described a binary grading system that uses a low magnification evaluation of the presence of necrosis, pattern of invasion, and amount of solid growth to divide endometrioid ECs into low- and high-grade tumors. The authors suggested that a tumor should be considered as high-grade when it exhibits at least two of the following features: i) more than 50% solid growth (without distinction of squamous from non-squamous epithelium); ii) a diffusely infiltrative, rather than expansive, growth pattern; and iii) tumor cell necrosis. The authors reported that both inter- and intraobserver agreements using the binary grading system were superior compared with the FIGO and nuclear grading systems. Scholten et al. (8) conducted a study to compare the reproducibility of FIGO grading system with the novel binary grading system proposed by Lax et al. (7); however, they found that the inter-observer agreement for both systems was moderate, with 70% and 73% agreement rates for the FIGO and binary grading systems, respectively. The authors proposed that if a simple architectural binary grading system that divides tumors into low- and high-grade based solely on the proportion of solid tumor growth (≤50% or ˃50%) was used in the grading of ECs, a much better agreement rate (85%) could be achieved. In another alternative binary grading system (low-grade vs. high-grade), Alkushi et al. (9) suggested that tumors should be considered high-grade in the presence of at least two of the following criteria: i) predominantly papillary or solid growth pattern, ii) mitotic index ≥6/10 high power fields, and iii) severe nuclear atypia. The authors reported that this system had more prognostic power than the three-tiered FIGO and binary system of Lax et al. (7) when applied to all tumors regardless of tumor histotype; however, the FIGO grading system was superior for prognostication when only endometrioid type ECs were considered.

Currently, none of these alternative systems has become widespread because it is not clear whether they would significantly improve the prognostic utility of the current method (10). Moreover, in a recent study comparing new binary systems with the existing three-tiered FIGO grading system, Guan et al. (11) demonstrated that the FIGO grading system using the nuclear criteria of Zaino et al. (4) was prognostically superior to the other systems, particularly in patients with endometrioid-type EC.

There are also some studies in the literature reporting that NG is more useful than FIGO grade in terms of predicting poor disease outcome (12,13). However, these trials are heterogeneous regarding tumor histotype. Non-endometrioid tumors including serous and clear cell histotypes are graded principally by nuclear features alone (3). Therefore, the association of NG with poor disease outcome in these trials, may in fact reflect the poor outcome of non-endometrioid tumors. On the other hand, in studies examining the tumors with endometrioid histology alone, architectural and FIGO grades have been mostly demonstrated to be prognostically superior to NG (4,6,11). 

In spite of sufficient data indicating prognostic validity of the current grading methods, there appears to be little exclusive data available on relationships of different grading systems specifically with LN involvement. Most of the data have focused on the risk of LN metastasis based on stratification of FIGO grade by myometrial invasion and/or tumor size, and usually demonstrated a dependent association (3,14,15,16). However, trials examining independent predictors of LN involvement by controlling the potential confounding factors such as myometrial invasion, tumor size, LVSI, and cervical involvement, generally failed to demonstrate a direct relationship between tumor grade and LN involvement (17,18). Consistent with previous studies, an independent relationship between any of the three grading systems and LN metastasis could not be demonstrated in our study. However, our findings should be cautiously interpreted because our study and its design have limitations including the relatively small sample size and single institutional nature, which bring inherent problems of selection and referral bias. The small sample size of our study might have caused a sampling error, limiting the power in detecting associations.

In conclusion, based on our results, nuclear grading is correlated with neither the architectural nor the FIGO grading systems. As opposed to architectural and FIGO grading systems, in which the LN involvement rates significantly differ in grade 2 level (AG 2 and FIGO grade 2), the differences in LN involvement rates in the nuclear grading system reach statistical significance only in the setting of tumor cells with grade 3 features. Therefore, “notable nuclear atypia” should be defined as NG 3. However, none of the grading systems is an independent predictor of LN metastasis.

## Figures and Tables

**Table 1:**
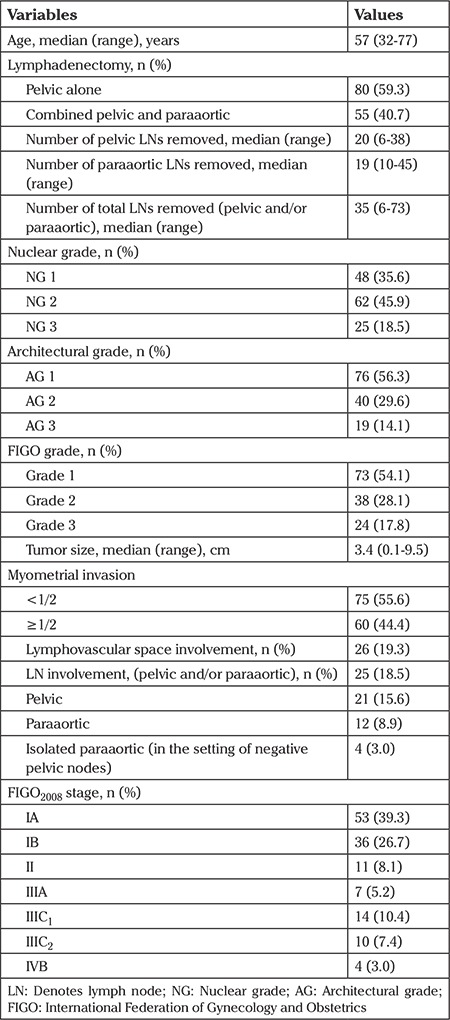
Characteristics of the patients

**Table 2:**
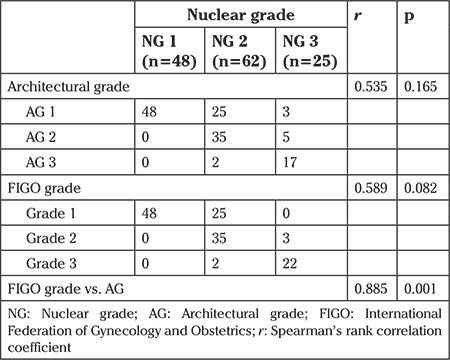
istribution of nuclear grades among each architectural and International Federation of Gynecology and Obstetrics grade, and correlations among grading systems

**Table 3:**
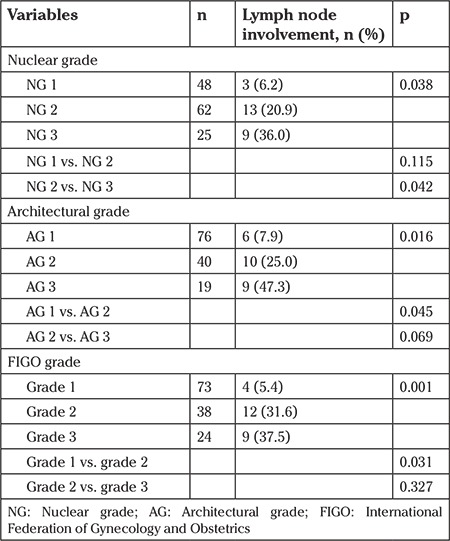
Lymph node involvement according to each grading system

**Table 4:**
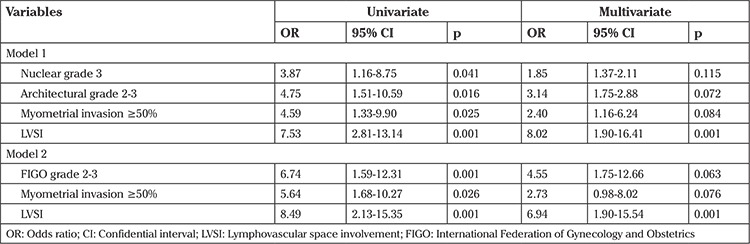
Relationships of grading systems with lymph node involvement
